# Optimization of Cultivation Substrate Formula and Key Physical Parameters for Domestication of *Floccularia luteovirens* by Response Surface Methodology

**DOI:** 10.3390/life16020355

**Published:** 2026-02-19

**Authors:** Xu Zhao, Siyuan Gou, Lihua Tang, Tongjia Shi, Zhiqiang Zhao, Wensheng Li, Yan Wan

**Affiliations:** 1 College of Food and Biological Engineering, Chengdu University, Chengdu 610106, China; 2 Institute of Urban Agriculture, Chinese Academy of Agricultural Sciences, Chengdu 610299, China; 3 Chengdu National Agricultural Science and Technology Center, Chengdu 610299, China; 4 The Edible Fungi Research Institute of Shanghai Academy of Agricultural Sciences, Shanghai 201403, China; 5 Zhuoni County Agricultural Technology Extension Station, Gannan 747600, China; 6 Key Laboratory of Coarse Cereal Processing, Ministry of Agriculture and Rural Affairs, College of Food and Biological Engineering, Chengdu University, Chengdu 610106, China

**Keywords:** *Floccularia luteovirens*, cultivation substrate formula, domestication and cultivation, response surface methodology, mycelial growth

## Abstract

*Floccularia luteovirens* is an edible and medicinal fungus with great development value on the Qinghai–Tibet Plateau, but its artificial domestication and cultivation are limited by the lack of systematic research on cultivation substrate formulas and key parameters. This study adopted the technical route of “preliminary screening—single-factor optimization—response surface collaborative optimization” to conduct research on the screening and optimization of its domestication cultivation substrate. Firstly, through the preliminary screening of 26 groups of formulas, a basic cultivation substrate formula with compatible complex nutrition and physical structure was determined. Secondly, single-factor experiments clarified that mixed sawdust was the optimal main substrate, corn flour was the optimal auxiliary substrate, the suitable substrate-to-water ratio was 1:1.6, and the suitable compactness was a substrate surface height of 12–12.5 cm (corresponding to a bulk density of 1.10–1.15 g/cm^3^ and a porosity of 60.6–63.3%). Finally, based on the response surface Box–Behnken model, with the main substrate, substrate-to-water ratio, and compactness as independent variables, and the total mycelial growth in 30 days as the response value, response surface optimization was performed to obtain the optimal formula: main substrate 76.002%, substrate-to-water ratio 1:1.721, and compactness 12.845 cm. Under these conditions, the mycelial growth reached 28.75 mm, which was highly consistent with the model’s predicted value (28.012 mm), and the constructed quadratic regression model showed excellent fitness (R^2^ = 0.9920, *p* = 0.0008). This study clarified the core influencing factors and adaptation mechanism of the cultivation substrate for *Floccularia luteovirens*, filled the research gap in the domestication cultivation substrate of this fungus, and provided basic technical parameters for its large-scale artificial cultivation.

## 1. Introduction

*F. luteovirens* is an excellent edible and medicinal fungus nurtured under the unique geographical and climatic conditions of the Qinghai–Tibet Plateau. As an important biological resource of the Qinghai–Tibet Plateau [1], it is recognized as one of the wild fungi with the highest development and utilization values. It is delicious in color, aroma and taste, and can be called a top-grade fungus. It was used as a tribute to the imperial court in the Qing Dynasty [2], and is rich in amino acids, containing 19 types [3]. As early as the Tang Dynasty, its medicinal value was recorded in the Four Medical Tantras. It has important effects such as stimulating appetite, preventing beriberi and neuritis, resisting influenza viruses [4], and promoting infant development. Extracts of its active ingredients can directly act as natural anti-tumor active substances that kill tumor cells, with such pharmacological functions [5,6].

As its value has been widely recognized, an increasing number of scholars have joined the research on *F. luteovirens*. Its economic, edible and medicinal values are self-evident [7], so the artificial domestication and cultivation of *F. luteovirens* has become an inevitable trend in industrial development. However, to date, research on the domestication of *F. luteovirens* has only focused on the screening of mother culture media [8,9,10,11,12], and no systematic research has been conducted on the formula and key parameters of domestication cultivation substrates. This critical gap leaves the fundamental requirements for its mycelial growth and substrate colonization poorly understood, thereby constituting a major obstacle to the entire domestication process and hindering the development of a viable artificial cultivation technology.

Based on this, this study is the first to carry out research on the screening and optimization of domestication cultivation substrates for *F. luteovirens*. Given the multifactorial nature of substrate optimization (involving nutritional components, moisture, and physical structure), we employed Response Surface Methodology (RSM). Using the technical route of “preliminary screening—single-factor optimization—response surface collaborative optimization”, this work clarifies the core components and physical conditions of the cultivation substrate suitable for mycelial growth. The study aims to fill the critical research gap in substrate optimization, thereby laying a foundational basis for constructing a large-scale artificial cultivation technology system for this fungus.

## 2. Materials and Methods

### 2.1. Tested Strain

*F. luteovirens* strain S4-1, preserved in the Institute of Urban Agriculture, Chinese Academy of Agricultural Sciences (CAAS).

### 2.2. Mother Culture Medium

Wheat flour 20 g, ammonium sulfate 3 g, potassium chloride 0.3 g, magnesium sulfate 0.3 g, potassium dihydrogen phosphate 0.3 g, agar 20 g, and distilled water 1000 mL.

### 2.3. Culture Conditions

LEAD-Tech constant temperature and humidity incubator, culture at a constant temperature of 25 °C in the dark, with natural CO_2_ and O_2_ levels, and humidity of 60%.

### 2.4. Determination of the Basic Formula for Cultivation Substrate

Referring to the cultivation substrate formulas and fermented substrates of existing commercially produced mushrooms (e.g., *Flammulina filiformis*, *Pleurotus ostreatus*, *Volvariella volvacea*, *Pleurotus eryngii*), a total of 26 preliminary screening cultivation substrate formulas were obtained (Table 1). The test tube culture method was adopted [13], and the preparation of preliminary screening cultivation substrates was as follows: the total weight of dry materials in each formula was 25.6 g, with a moisture content of 60–65% (determined by the hand-squeezing method [14]). After mixing the dry substrates and water thoroughly, the mixture was equally distributed into test tubes (18 × 200 mm) with a substrate height of 13 cm. The pH was natural, and the substrates were sterilized at 121 °C and 0.15 MPa for 1.5 h.

Mother culture Petri dishes with vigorous mycelial growth were selected, and 9 mm mycelial plugs were cut along the edge of the colonies using a puncher, then inoculated into the cultivation substrate test tubes. Each test tube was inoculated with 4 mycelial plugs, and each treatment had 4 replicates. The inoculated test tubes were cultured at 25 °C in a constant-temperature incubator in the dark, with observations recorded every 24 h until the final determination of the basic formula. The basic formula for *F. luteovirens* was determined based on indicators including mycelial plug germination time, germination uniformity, mycelial colonization, substrate colonization rate, and mycelial growth vigor (one optimal basic formula was selected). Mycelial growth vigor was classified into 4 grades, denoted by +, ++, +++, and ++++ respectively: + indicates white and sparse mycelia; ++ indicates white and relatively sparse mycelia; +++ indicates pure white and relatively dense mycelia; +++++ indicates pure white and dense mycelia [15].

### 2.5. Single-Factor Experiment of the Basic Formula for Cultivation Substrate

Based on the basic formula determined by preliminary screening, a single-factor experiment was conducted to screen the main substrates, auxiliary materials, substrate–water ratio, and compactness.

Basic formula: Mixed sawdust 30%, cottonseed hulls 25%, corn cob 18%, wheat bran 15%, corn flour 5%, soybean meal 5%, calcium superphosphate 1%, gypsum 1%.

#### 2.5.1. Screening of Main Substrates

While keeping the proportions of auxiliary materials and other growth factors in the basic formula unchanged, the experimental design for main substrate screening is as follows (Table 2):

(1) Single-factor extreme-level groups: Three control groups were set up, with each group containing only one main substrate (mixed sawdust, cottonseed hulls, or corn cob) accounting for 73% of the total medium weight and the other two main substrates accounting for 0%.

(2) Two-factor interaction groups: Three experimental groups were designed, where each group used only two main substrates (the third main substrate accounted for 0%), and their total proportion was adjusted to 100% of the main substrate share (i.e., 73% of the total medium weight).

(3) Three-factor synergy groups: All three main substrates were guaranteed to occupy a certain proportion (none was 0%). In each group design, the proportions of the three main substrates were adjusted to achieve a dynamic balance of “one increases while the others decrease”.

The basic formula was used as the control (CK). The preparation of cultivation substrates for main substrate screening, as well as other conditions and treatments, were consistent with those described in Section 2.4. Each treatment had 4 replicates. On the 15th day of cultivation after inoculation, the first line marking and mycelial growth measurement were performed. Subsequently, mycelial growth was measured every 5 days for a total of 4 measurements, and a termination line was marked on the 30th day of cultivation. The average total growth and average growth rate were statistically analyzed and calculated [16]. Meanwhile, the mycelial color and growth vigor were observed and recorded, and the grading standard for mycelial growth vigor was the same as that in Section 2.4. The calculation formula for the average mycelial growth rate (mm/d) is:

Average mycelial growth rate = (Measurement value of the second mycelial growth − Measurement value of the first mycelial growth)/Time interval between the two measurements.

**Table 2:** Experimental design for screening of main substrates.

Serial Number	Main Substrates (%)			Auxiliary Materials (%)			Calcium Superphosphate (%)	Gypsum (%)
	Mixed Sawdust	Cottonseed Hulls	Corn Cob	Wheat Bran	Corn Flour	Soybean Meal		
CK	30	25	18	15	5	5	1	1
1	73	0	0	15	5	5	1	1
2	0	73	0	15	5	5	1	1
3	0	0	73	15	5	5	1	1
4	39	34	0	15	5	5	1	1
5	42.5	0	30.5	15	5	5	1	1
6	0	40	33	15	5	5	1	1
7	36.5	21.75	14.75	15	5	5	1	1
8	24.25	36.5	12.25	15	5	5	1	1
9	20.75	15.75	36.5	15	5	5	1	1

#### 2.5.2. Screening of Auxiliary Materials

While keeping the proportions of main substrates and other growth factors in the basic formula unchanged, the experimental design for auxiliary material screening is as follows (Table 3):

(1) Single auxiliary material with extreme proportion: Three high-level groups for a single auxiliary material were set up. The proportion of one auxiliary material was increased by 25% in each group, while the proportions of the other two auxiliary materials were set to 0%.

(2) Two auxiliary materials with synergistic combination: The total amount of auxiliary materials was kept unchanged, the proportion of the third auxiliary material was set to 0%, and the proportions of the remaining two auxiliary materials were adjusted simultaneously.

The optimal formula selected from main substrate screening was used as the control (CK). The preparation of cultivation substrates for auxiliary material screening, as well as other conditions and treatments, were consistent with those described in Section 2.4. The index determination and analysis methods were the same as those in Section 2.5.1.

**Table 3:** Experimental design for screening of auxiliary materials.

Serial Number	Main Substrates (%)	Auxiliary Materials (%)			Calcium Superphosphate (%)	Gypsum (%)
CK	73	15	5	5	1	1
1	73	25	0	0	1	1
2	73	0	25	0	1	1
3	73	0	0	25	1	1
4	73	12.5	12.5	0	1	1
5	73	12.5	0	12.5	1	1
6	73	0	12.5	12.5	1	1

#### 2.5.3. Screening of Substrate–Water Ratio

A total of 22 substrate–water ratio treatments were set up with a range of 1:0.5 to 1:2.6 and an interval of 0.1, namely 1:0.5, 1:0.6, 1:0.7, 1:0.8, 1:0.9, 1:1.0, 1:1.1, 1:1.2, 1:1.3, 1:1.4, 1:1.5, 1:1.6, 1:1.7, 1:1.8, 1:1.9, 1:2.0, 1:2.1, 1:2.2, 1:2.3, 1:2.4, 1:2.5, and 1:2.6.

The optimal formula selected from auxiliary material screening was used as the control (CK). The preparation of cultivation substrates for substrate–water ratio screening, as well as other conditions and treatments, were consistent with those described in Section 2.4. The index determination and analysis methods were the same as those in Section 2.5.1.

#### 2.5.4. Screening of Compactness

A total of 9 compactness treatments were set up with a substrate surface height of 10–14 cm in test tubes and an interval of 0.5 cm, namely 10 cm, 10.5 cm, 11 cm, 11.5 cm, 12 cm, 12.5 cm, 13 cm, 13.5 cm, and 14 cm. The preparation of cultivation substrates for compactness screening, as well as other conditions and treatments, were consistent with those described in Section 2.4. The index determination and analysis methods were the same as those in Section 2.5.1.

Compactness is an intuitive and porosity-related parameter. Therefore, after determining the appropriate compactness range, the modified saturated water drainage method was adopted [17]. Specifically, the cultivation substrates were subjected to saturated water absorption, drying, and weighing, with 3 replicates. The bulk density and porosity corresponding to the optimal compactness of the cultivation substrates were calculated using the following formulas:

Bulk density formula: ρ = m/V

Porosity formula: P = [(M − m)/(ρ_water_ × V)] × 100%; V = πr^2^h

Explanation of symbols in the formulas:

m: Dry mass of the cultivation substrates (g);
V: Total volume of the cultivation substrates (cm^3^);
M: Saturated mass of the cultivation substrates (g);
ρ_water_: Density of water (≈1 g/cm^3^); π: ≈3.14;
r: Radius of the test tube (0.9 cm, corresponding to the test tube specification of 18 × 200 mm);
h: Substrate surface height in the test tube (cm), which represents the compactness level.

### 2.6. Response Surface Optimization Experiment of Cultivation Substrate Formula

Based on the results of single-factor experiments, the Box–Behnken model was used to optimize the suitable cultivation substrate formula affecting the growth of *F. luteovirens* strain S4-1. Design-Expert 13 software was employed, with the total mycelial growth (mm) after 30 days of cultivation as the response value. Referring to Reference [18], the Box–Behnken experiment was conducted in accordance with its design requirements. Three independent variables were selected: main substrates, substrate–water ratio, and compactness, denoted as A, B, and C respectively. The low, medium, and high levels of each independent variable were coded as −1, 0, and 1 (Table 4).

### 2.7. Data Processing and Statistical Analysis

Microsoft Excel 2021 (Microsoft Corp, Redmond, WA, USA) was used to sort out the experimental data, and SPSS 26.0 (IBM Corp, Armonk, NY, USA) was employed for one-way analysis of variance (ANOVA). Origin 2017 software was utilized for data visualization and graph plotting. The Box–Behnken response surface experiment was designed, statistically analyzed, and graphed using Design-Expert 13 software.

## 3. Results

### 3.1. Experimental Results and Analysis of Basic Formula Determination for Cultivation Substrate via Preliminary Screening

Significant differences in mycelial growth of *F. luteovirens* were observed among the 26 preliminary screening formulas. After inoculation, only the mycelial blocks of Formulas 16 and 17 failed to germinate; although the mycelial blocks of the other formulas all germinated, there were obvious differences in mycelial growth vigor. Meanwhile, the germination time of mycelial blocks varied among different cultivation substrate groups: Formulas 1, 2, 4, 5, and 6 germinated 2 days after inoculation; Formulas 3, 7, 8, 9, 12, 14, 18, 19, 20, 21, 23, and 25 germinated 3 days after inoculation; Formulas 10, 11, 13, 15, 22, 24, and 26 germinated 4 days after inoculation.

After germination, only Formulas 1, 4, 5, 6, 7, 12, 25, and 26 achieved mycelial colonization and substrate utilization. Among these, Formulas 1, 5, 7, 25, and 26 began to colonize and grow by utilizing the substrate 10 days after inoculation; however, Formula 1 grew too slowly, while Formula 5 showed continuous growth with increasing vigor. Formula 6 started colonization and substrate utilization 15 days after inoculation, and Formula 12 began 20 days after inoculation. The latest colonization was observed in Formula 4, which started 25 days after inoculation (Table 5).

Based on a comprehensive analysis of mycelial growth vigor, Formula 5 was ultimately selected as the basic formula for subsequent experiments. Its composition is as follows: mixed sawdust 30%, cottonseed hulls 25%, corn cob 18%, wheat bran 15%, corn flour 5%, soybean meal 5%, calcium superphosphate 1%, and gypsum 1%.

### 3.2. Experimental Results and Analysis of Single-Factor Experiments on the Basic Formula for Cultivation Substrate

#### 3.2.1. Effects of Different Main Substrates on Average Mycelial Growth Rate and Total Mycelial Growth

Different main substrate-based cultivation formulas had significant effects on the mycelial growth of *F. luteovirens*. As shown in Table 6, mycelia could grow in all nine main substrate formulas, and the average growth rate showed a trend of first increasing and then decreasing during the 30-day cultivation period. The mycelial growth rate of almost all formulas (including CK) reached a peak on the 25th day, followed by varying degrees of decline on the 30th day, indicating that the mycelia entered a vigorous growth stage around the 25th day and then growth slowed down.

Formula 1 performed the best: its average growth rate on the 25th day reached 1.000 ± 0.163 mm/d, which was significantly higher than all other treatments (with a significance marker of “a”), and the mycelial growth vigor was rated “++++” (white and dense), making it the optimal formula. Formulas 6 and 3 also showed relatively high growth rates in the mid–late growth stage (20–25 days), reaching 0.850 mm/d and 0.850/0.550 mm/d respectively, with mycelial growth vigor rated “++++” for both, serving as excellent alternative formulas. Formula 2 had the lowest growth rate at all time points, showing significant differences from most other formulas, indicating that its substrate composition may not be suitable for the mycelial growth of *F. luteovirens*.

**Figure 1:**
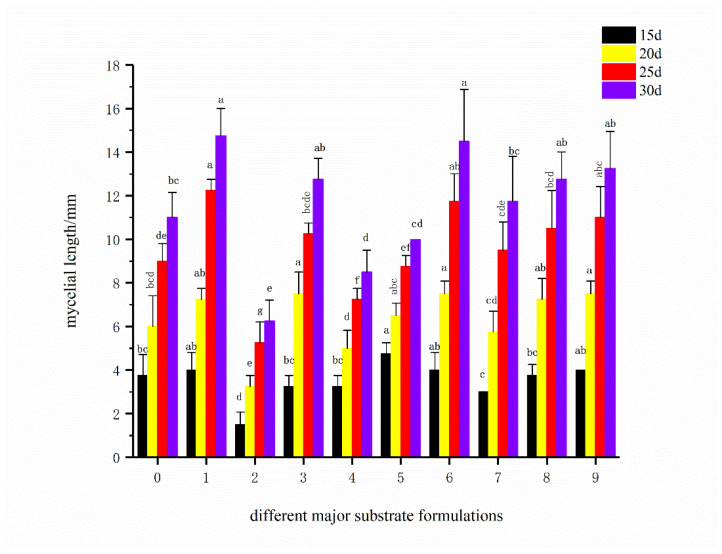
Total Mycelial growth at different cultivation times under different main substrate formulas (the values 0–9 on the horizontal axis correspond to the main substrate formulas CK-9).

As shown in Figure 1, under different main substrate formulas, the total mycelial growth of *F. luteovirens* significantly increased with the extension of cultivation time. After 30 days of cultivation, the total mycelial length of most formulas exceeded 10 mm, indicating that all formulas could support the basic growth of mycelia. The mycelial growth roughly showed the typical S-shaped curve characteristic of “slow—fast—slow”: specifically, it entered the rapid growth stage from 15 to 25 days, and the growth rate slowed down slightly from 25 to 30 days.

Based on a comprehensive analysis of the final total mycelial growth at 30 days, Formula 1 performed the best, with a total mycelial length close to or reaching 18 mm, which was significantly higher than that of most other formulas. This indicates that the substrate composition of Formula 1 is superior to CK, making it the optimal candidate. Formulas 6 and 3 also exhibited good supportive capacity, with total mycelial growth of approximately 14 mm and 12 mm at 30 days, respectively, serving as excellent alternative formulas. Formula 2 had the poorest final mycelial growth (only close to 6 mm).

Considering the performance of average growth rate and total mycelial growth throughout the 30-day cultivation period, Formula 1 was determined to be the most suitable cultivation substrate formula for the mycelial growth of *F. luteovirens*, featuring fast growth rate and good growth vigor. Formulas 3 and 6 were identified as good alternative formulas, while Formula 2 showed the worst mycelial growth. Thus, Formula 1 from main substrate screening was selected as the iterative basic formula for subsequent auxiliary material screening experiments.

#### 3.2.2. Effects of Different Auxiliary Materials on Average Mycelial Growth Rate and Total Mycelial Growth

*F. luteovirens* could grow in all different auxiliary material formulas. As shown in Table 7, overall, the mycelial growth rate showed a trend of first increasing and then decreasing with the extension of cultivation time. During 20–25 days of cultivation, the growth rate of most formulas reached a peak. Among them, Formula 2 maintained a relatively high average growth rate at all time points, especially when it reached 1.050 mm/d on the 20th day and remained at the highest level of 0.850 mm/d on the 30th day. Formulas 4 and CK also exhibited fast growth rates in the early–mid growth stage (20th day), with 1.000 mm/d and 0.850 mm/d respectively. In contrast, the growth rate of Formula 3 was consistently significantly lower than that of other formulas, and its rate was extremely low after the 20th day (0.200–0.250 mm/d).

**Figure 2:**
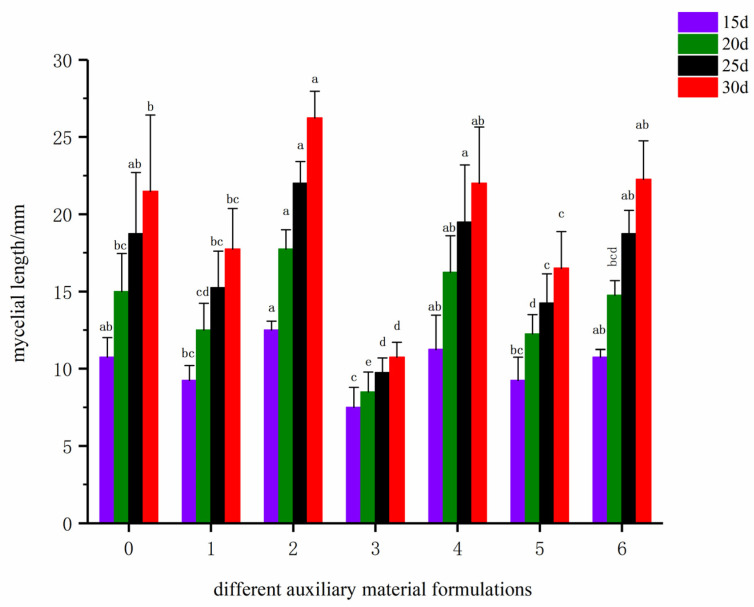
Total mycelial growth at different cultivation times under different auxiliary material formulas (the values 0–6 on the horizontal axis correspond to the main substrate formulas CK-6).

As can be seen from Figure 2, the total mycelial growth showed a cumulative effect with the extension of cultivation time. The total mycelial length of all formulas increased significantly over time. After 30 days of cultivation, Formula 2 had the highest total mycelial growth, close to 25 mm. Formulas 6 and CK followed, with their total mycelial lengths also reaching relatively high levels. The total mycelial growth of Formula 3 was consistently the lowest among all formulas, only about 12 mm on the 30th day, which was significantly lower than other treatments.

Based on a comprehensive analysis of the average growth rate and total mycelial growth, auxiliary material Formula 2 was determined to be the optimal choice for cultivating *F. luteovirens*, as it could simultaneously support rapid mycelial growth and maximum growth yield. Formula 6 was also a feasible alternative formula, while Formula 3 was completely unsuitable for the cultivation of this fungus. Thus, Formula 2 from auxiliary material screening was selected as the iterative basic formula for subsequent substrate–water ratio screening experiments.

#### 3.2.3. Effects of Different Substrate–Water Ratios on Average Mycelial Growth Rate and Total Mycelial Growth

Data in Table 8 show that the average mycelial growth rate exhibited regular changes depending on cultivation time and substrate–water ratio. At 15 days, the growth rates of substrate–water ratios 1:1.6 (0.965 mm/d) and 1:1.7 (0.933 mm/d) were significantly higher than those of all other formulas (both belonging to significance group “a”), indicating that moderate water content was most favorable for early mycelial germination. At 20 days, the growth rate of substrate–water ratio 1:1.8 reached the maximum value of 1.050 mm/d (group “A”). Meanwhile, the growth rates of substrate–water ratios 1:2.2 and 1:2.3 were both 1.000 mm/d (both belonging to group “AB”), showing no significant difference from 1:1.8 but significantly higher than most formulas, thus performing excellently at this stage. At 25 days, the growth rate of substrate–water ratio 1:2.3 maintained the highest level of 1.000 mm/d (group “ab”), and that of 1:2.2 was 0.900 mm/d (group “abcd”), with both remaining at the leading level. At 30 days, the growth rate of substrate–water ratio 1:1.6 was the highest (0.850 mm/d, group “A”), demonstrating good sustained growth capacity.

From the perspective of dynamic growth rate, different formulas showed respective advantages at different growth stages: substrate–water ratio 1:1.6 performed best at 15 and 30 days, indicating its ability to support rapid mycelial colonization and maintain late-stage growth momentum. Ratios 1:1.8, 1:2.2, and 1:2.3 had obvious advantages during 20–25 days, suggesting that slightly higher water content could better promote rapid mycelial extension at this stage.

**Figure 3:**
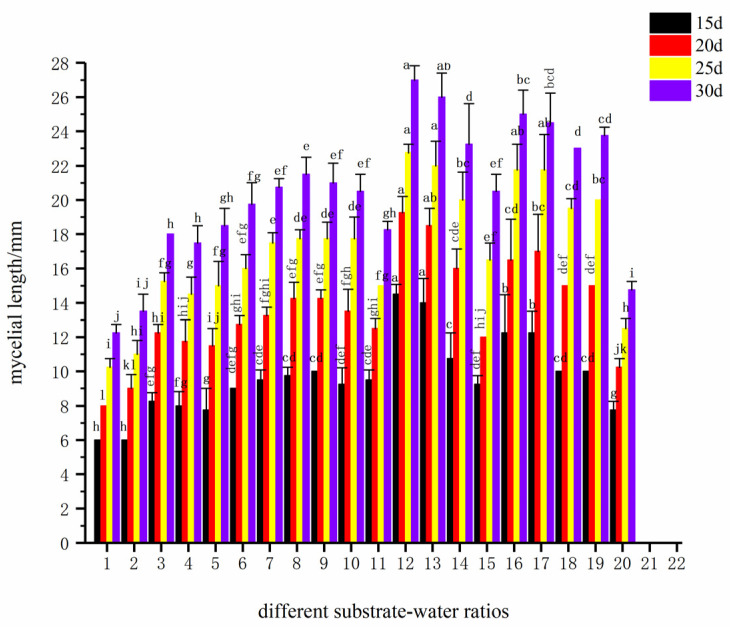
Total mycelial growth at different cultivation times under different substrate–water ratio formulas (the values 1–22 on the horizontal axis correspond to the substrate–water ratios ranging from 1:0.5 to 1:2.6).

Figure 3 reflects the cumulative effect of total mycelial growth, which is highly consistent with the growth rate data. After 30 days of cultivation, the total mycelial growth of substrate–water ratio 12 (corresponding to 1:1.6) was significantly higher than that of all other formulas, supporting the maximum mycelial biomass accumulation. At 20 and 25 days, the total mycelial growth of substrate–water ratios 16 (1:2.2) and 17 (1:2.3) also reached very high levels, which was completely consistent with their high growth rates at these stages in Table 8. For formulas with substrate–water ratio ≤1:0.8 (low water content) and ≥1:2.4 (high water content), their total mycelial growth was consistently significantly lower, indicating that water stress inhibited mycelial growth.

Based on a comprehensive analysis of the results from the table and figure, substrate–water ratio 1:1.6 supported the highest mycelial growth, indicating that its water condition achieved an optimal balance throughout the entire growth cycle, meeting both growth requirements and maintaining good air permeability. Although ratios 1:2.2 and 1:2.3 converted their rapid growth during 20–25 days into considerable mycelial growth, their final growth was lower than that of 1:1.6. This may be due to the slightly higher water content in the later stage slightly affecting air permeability, resulting in insufficient late-stage growth momentum. Therefore, the optimal substrate–water ratio for the cultivation substrate of *F. luteovirens* was ultimately determined to be 1:1.6, which was selected as the iterative basic formula for subsequent compactness screening experiments.

#### 3.2.4. Effects of Different Substrate Compactness Levels on Average Mycelial Growth Rate and Total Mycelial Growth

Data in Table 9 show that substrate surface height (representing compactness) had a significant effect on the average mycelial growth rate of *F. luteovirens*, and this effect was time-specific. At 15 days, the average mycelial growth rate was the highest (0.715 mm/d) when the substrate surface height was 12.5 cm, which belonged to the most significant group (letter “a”) together with the treatments of 12.0 cm, 13.0 cm, and 13.5 cm. This indicates that moderate compactness was most favorable for early mycelial germination. The growth rate was the lowest (0.565 mm/d) when the substrate surface height was 11.0 cm.

At 20 days, the growth rates were the highest (both 0.900 mm/d) for substrate surface heights of 12.0 cm and 12.5 cm, while the growth rate was significantly the lowest (0.650 mm/d, group “B”) when the height was 14.0 cm. During 25–30 days, the advantages remained concentrated in the moderate compactness range: at 25 days, the growth rates were the highest (0.800–0.850 mm/d, group “a”) for 12.0 cm, 12.5 cm, and 13.5 cm. By 30 days, there was no significant difference among all treatments (all belonging to group “A”), but the treatment with 11.5 cm showed the highest average value (1.100 mm/d). However, its standard deviation was relatively large (±1.013), so the data stability is questionable.

Comprehensive analysis of the entire cultivation period revealed that formulas with a substrate surface height of 12.0 cm to 13.5 cm (i.e., moderate compactness) could support mycelia to maintain a high and stable growth rate at all stages. This indicates that this compactness range can well balance the air permeability and water retention of the substrate, making it most suitable for mycelial growth. When the substrate surface height was reduced to 11.0 cm (more compact substrate), mycelial germination at 15 days was significantly inhibited (group “d”). When the height was increased to 14.0 cm (looser substrate), the mycelial growth rates at 20 and 25 days were both significantly the lowest (groups “B” and “b”). This suggests that either excessively compact or excessively loose substrate would impose stress on mycelial growth.

**Figure 4:**
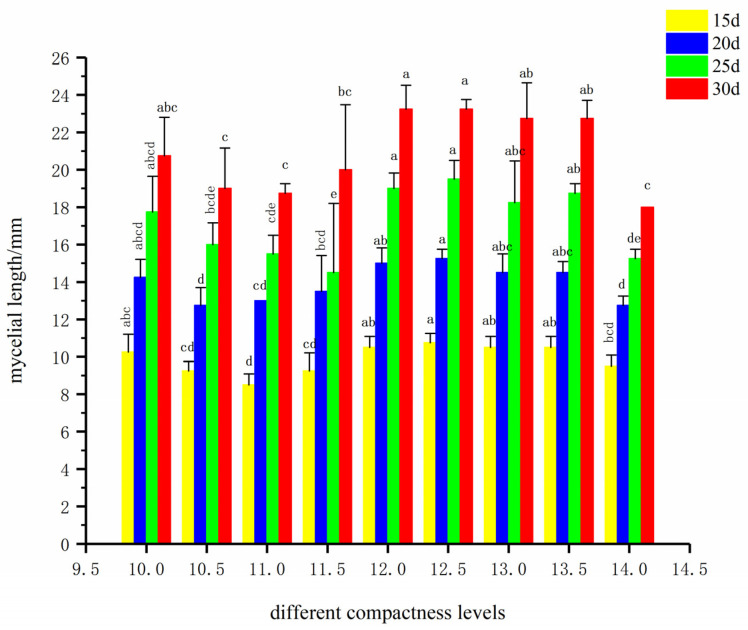
Total mycelial growth at different cultivation times under different substrate compactness formulas.

The results in Figure 4 are highly complementary to those in Table 9. After 30 days of cultivation, the treatments with substrate surface heights of 12.0 cm and 12.5 cm supported the maximum total mycelial growth (close to 23 mm). At all cultivation time points (15, 20, 25, and 30 days), the mycelial lengths corresponding to substrate surface heights of 12.0 cm and 12.5 cm were consistently at the highest or second-highest level. The treatment with 14.0 cm showed significantly lower mycelial growth at all stages.

Based on a comprehensive analysis of the results from the table and figure, the optimal substrate surface height (compactness) for the formula was determined to be 12.0–12.5 cm. According to the compactness screening results, the bulk density and porosity of the culture tubes with substrate surface heights of 12.0 cm and 12.5 cm were calculated using the formula in Section 2.4 via the saturated drainage method, and the specific results are shown in Table 10.

### 3.3. Results and Analysis of Response Surface Optimization Experiments

#### 3.3.1. Regression Fitting and Significance Analysis of Mycelial Growth Yield of *Floccularia luteovirens*

Taking main substrate (A), substrate–water ratio (B), and substrate compactness (C) as independent variables, and mycelial growth yield (Y) as the response value, a Box–Behnken central composite design was adopted. Groups 1–12 were factorial experiments, and the remaining groups were central experiments. Among the 15 experimental groups, the mycelial growth yield ranged from 17.5 to 27 mm, as shown in Table 11.

Although the quadratic regression model initially constructed based on the results in Table 11 was overall significant (*p* = 0.0090), it exhibited a high sum of squared residuals (16.33). Additionally, the key main effect terms—main substrate (A) and substrate–water ratio (B)—did not reach a significant level (*p* > 0.05), indicating potential interference from outliers in the model. In this study, a standardized residual test was conducted on the data of all experimental groups in accordance with the statistical criteria for outlier identification. The results showed that the absolute value of the standardized residual for Group 6 (73% main substrate, substrate-to-water ratio of 1:1.6, compactness of 11 cm) far exceeded the critical value of ±3, classifying it as an extreme statistical outlier. This value deviated from the normal distribution law of the data and thus had a valid statistical basis for exclusion. Under this condition, the mycelial growth yields of other replicate experiments ranged from 17.5 to 26.5 mm, while the measured value of Group 6 (9.7 mm) significantly deviated from the normal range. This deviation may have been caused by non-systematic errors during operation or measurement (it is speculated that the activity of the mycelial mass was impaired during inoculation).

To eliminate the interference of extreme outliers on the fitting accuracy of the model and improve the reliability and interpretability of the regression model, this set of outliers was removed before model reconstruction. After removing the outliers and rebuilding the model, the statistical performance of the model was significantly improved. To establish the relationship between the response value (mycelial growth yield) and independent variables, quadratic polynomial regression fitting was performed on the experimental results (excluding the data of Group 6), yielding the following quadratic multiple regression equation:

Y = −161.856 − 0.361A − 17.675B + 32.405C + 0.975AB + 0.265AC + 13.75BC − 0.029A^2^ − 66.25B^2^ − 3.0C^2^

To evaluate the reliability and accuracy of the model, analysis of variance (ANOVA) was conducted. Table 12 presents the results of the significance test for the regression model. The model’s F-value was 55.20 with *p* < 0.05, indicating that the model was significant and the experimental method was reliable. The lack-of-fit term was not significant (*p* = 0.7475 > 0.05), demonstrating good fitting between the model and the measured values with minimal interference from factors other than the experimental variables. The R^2^ of the regression equation was 0.9920, meaning 99.2% of the variability could be explained by the regression model, confirming excellent agreement between the equation and actual conditions. The adjusted coefficient of determination (adjusted R^2^) was 0.9740, further indicating that the model still maintained extremely high explanatory power after controlling for the number of independent variables, with no overfitting observed.

All terms investigated in the model reached an extremely significant level (*p* < 0.01). The *p*-values of the three single factors—main substrate (A), substrate–water ratio (B), and substrate compactness (C)—were 0.0055, 0.0039, and 0.0027, respectively, indicating that each factor exerted an extremely significant independent effect on mycelial growth yield. The *p*-values of all interaction terms (AB, AC, BC) and quadratic terms (A^2^, B^2^, C^2^) were less than 0.01, among which the interaction term BC was the most significant (*p* = 0.0009). This clearly reveals the presence of complex nonlinear relationships and interaction effects among the parameters, confirming that the use of response surface methodology for optimization was necessary and accurate.

#### 3.3.2. Response Surface Analysis and Determination of Optimal Levels of Various Factors

**Figure 5:**
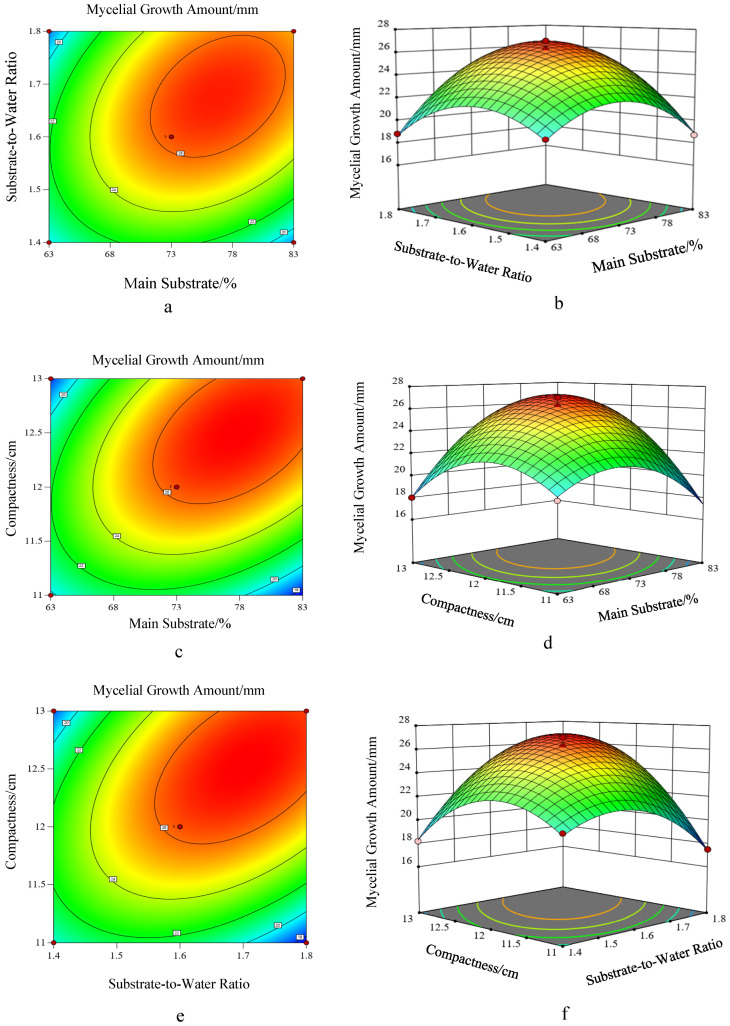
Response surface contour and 3D plots of the effects of interaction between factors on mycelial growth yield of *Floccularia luteovirens* ((**a**,**b**): Contour and 3D plots of the interaction between main substrate and substrate–water ratio; (**c**,**d**): Contour and 3D plots of the interaction between main substrate and substrate compactness; (**e**,**f**): Contour and 3D plots of the interaction between substrate–water ratio and substrate compactness).

Response surface contour plots are presented in a two-dimensional plane, illustrating the effects of combinations of two factors on the response value and the distribution of the optimal region through contour lines with precise and clear expression. Response surface 3D plots, on the other hand, adopt a three-dimensional format to intuitively display the interaction effects between factors. Figure 5 shows the response surface contour plots and 3D plots of the effects of interaction between various factors on the mycelial growth yield of *Floccularia luteovirens*.

The contour lines are elliptical, indicating the presence of significant interaction effects between the two factors. The steeper the response surface curve, the more significant the effect of the factors on the response value; the gentler the trend, the smaller the impact of the factors on the results.

It can be seen from the response surface 3D plots and contour plots in Figure 5 that under the interaction of main substrate (A) and substrate–water ratio (B) (Figure 5a,b), the mycelial growth yield underwent significant changes as the substrate–water ratio varied within the range of 1:1.4–1:1.8, resulting in a notably steep surface. The growth trend first increased rapidly and then tended to level off. When the substrate–water ratio was fixed and the main substrate content varied between 73% and 83%, the variation range in the 3D plot was more pronounced compared to the trend of mycelial growth yield when the main substrate was fixed and the substrate–water ratio was used as the independent variable. Comprehensive analysis indicates that under the interaction of these two factors, the effect of the main substrate on mycelial growth yield was more significant than that of the substrate–water ratio.

Under the interaction of main substrate (A) and substrate compactness (C) (Figure 5c,d), the mycelial growth yield first increased rapidly and then gradually decreased as the main substrate content varied from 63% to 83% and the substrate compactness adjusted between 11 cm and 13 cm. Both the contour plots and 3D surface plots show that the mycelial growth yield reached a high level under specific combinations of main substrate and compactness, with the color gradient from blue to red indicating a gradual increase in growth yield. The surface morphology reveals that changes in the main substrate had a more significant impact on mycelial growth yield: under the same compactness condition, adjusting the main substrate content caused substantial fluctuations in growth yield, while the effect of compactness changes was relatively gentle. The mycelial growth yield peaked when the main substrate was within the optimal range (approximately 73–78%) and the substrate compactness was around 12 cm. Comprehensive analysis demonstrates that in their interaction, the main substrate was the dominant factor affecting mycelial growth yield, while substrate compactness exerted a certain synergistic regulatory effect but with a relatively weaker influence.

Under the interaction of substrate–water ratio (B) and substrate compactness (C) (Figure 5e,f), the mycelial growth yield exhibited regular changes. In the 3D plot, the surface showed a distinct arch shape. When the substrate compactness was fixed and the substrate–water ratio varied within the range of 1:1.4–1:1.8, the mycelial growth yield underwent substantial changes, resulting in a notably steep surface along the substrate–water ratio direction (*X*-axis). The growth trend was characterized by a rapid initial increase followed by a slow decrease.

In contrast, when the substrate–water ratio was fixed and the substrate compactness adjusted between 11 cm and 13 cm, the variation range of the 3D plot along the substrate compactness direction (*Y*-axis) was relatively gentle compared to the trend observed when the compactness was fixed and the substrate–water ratio served as the independent variable. This analysis was further validated by the shape of the contour lines, which were distinctly elliptical with their major axis roughly parallel to the substrate–water ratio axis. This intuitively indicates that changes in the substrate–water ratio had a more significant impact on mycelial growth yield.

Comprehensive analysis demonstrates that under the interaction of these two factors, the effect of the substrate–water ratio on mycelial growth yield was more significant than that of substrate compactness.

#### 3.3.3. Validation of the Regression Model

Optimization was performed through response surface analysis and the numerical optimization technology provided by the software. The optimal formula conditions for *F. luteovirens* culture substrate were determined as follows: main substrate 76.002%, substrate–water ratio 1:1.721, and substrate compactness 12.845 cm. The predicted mycelial growth yield was 28.012 mm.

Experiments were conducted using the optimized factors, and the actual mycelial growth yield of *F. luteovirens* reached 28.75 mm, which was close to the predicted value of the model. Thus, the model was verified to be reliable.

## 4. Discussion

As a rare endemic edible and medicinal fungus on the Qinghai–Tibet Plateau, *F. luteovirens* possesses extremely high industrial development value due to its rich amino acid composition and significant pharmacological activities such as anti-tumor [19] and anti-influenza [20,21,22]. Artificial large-scale cultivation is the core path to break reliance on wild resources and achieve sustainable utilization [23,24]. Previous relevant domestication studies have been limited to the selection of mother culture media, and no systematic research has been conducted on the nutritional ratio and physical properties of domesticated culture substrates, resulting in key shortcomings in the artificial cultivation technology system. In this study, through the technical route of “preliminary screening—single-factor optimization—response surface synergistic optimization”, the optimal formula and key influencing factors of the culture substrate for *F. luteovirens* strain S4-1 were clarified for the first time. This provides core technical support for its artificial domestication and cultivation, and also offers a reference paradigm for the development of culture substrates for similar plateau fungi.

The preliminary screening results of this experiment showed that there were significant differences in the adaptability of 26 culture substrate formulas to the mycelial growth of *F. luteovirens*. Only eight formulas achieved mycelial colonization and continuous growth. Formulas 16 and 17 failed to germinate completely possibly due to an imbalanced carbon-to-nitrogen ratio and insufficient air permeability caused by a high proportion of dry cow dung powder. Most formulas exhibited failure to colonize or death in the later stage, which may be attributed to unbalanced nutrition or incompatible physical structure. The ultimately selected basic formula (mixed sawdust 30% + cottonseed hulls 25% + corn cob 18% + wheat bran 15% + corn flour 5% + soybean meal 5% + superphosphate 1% + gypsum 1%) laid a scientific foundation for subsequent single-factor optimization.

The single-factor optimization results further revealed the regulatory mechanisms of each component and physical parameter of the culture substrate on mycelial growth. Regarding the main substrate, the single main substrate formula with 73% mixed sawdust performed optimally, achieving a total mycelial growth of nearly 15 mm in 30 days and a peak growth rate of 1.000 ± 0.163 mm/d. However, mixed main substrate combinations exhibited inferior growth performance compared to single mixed sawdust due to imbalanced particle size distribution or competitive carbon source metabolism, indicating that *F. luteovirens* has a strong selectivity for main substrate types. This result is highly consistent with the findings of Wang et al. [25] in their study on *Lentinus edodes*. Their research revealed that hardwood sawdust, as a core main substrate derived from broad-leaved trees, provides abundant and easily degradable lignin, cellulose and hemicellulose for the growth of wood-rotting fungi, serving as a pivotal carbon source carrier to underpin the basic mycelial growth. The cultivation system with hardwood sawdust as the main substrate can significantly improve the efficiency of mycelial colonization and the stability of mycelial growth.

In the auxiliary substrate screening, the single high-proportion formula with 25% corn flour was optimal, with a total mycelial growth exceeding 25 mm in 30 days and a growth rate of 1.050 ± 0.191 mm/d at 20 days. This is presumably attributed to its easily digestible starch and proteins that can be rapidly absorbed by mycelia [26], meeting the nutritional requirements during the rapid growth stage. The nutritional supply efficiency of mixed auxiliary substrate combinations was lower than that of a single high-quality auxiliary substrate, which is consistent with previous research conclusions on the preference of fungi for easily digestible nitrogen sources [27,28]. Lin et al. [29] also confirmed in their auxiliary substrate screening experiment for *Ganoderma lucidum* cultivation with hardwood sawdust that corn flour is the optimal type of auxiliary substrate in the hardwood sawdust-based main substrate system. It not only supplements readily available carbon and nitrogen sources for the growth of fungal mycelia but also adjusts the carbon–nitrogen ratio of the cultivation substrate to an optimal range, which significantly accelerates the mycelial bag-running speed and increases the primordium formation rate and fruiting body yield. Moreover, the yield-promoting effect of corn flour is significantly superior to that of other auxiliary substrates such as wheat bran, rice bran and rice straw powder. The growth advantage of *F. luteovirens* in the single auxiliary substrate system with corn flour observed in this study provides a cross-species verification with the findings of the *Ganoderma lucidum* study, indicating that the “main substrate + auxiliary substrate” combination of hardwood sawdust and corn flour is a highly efficient nutritional matching mode for the cultivation substrates of wood-rotting fungi. By supplementing readily available nutrients and optimizing the nutritional structure, corn flour can effectively address the deficiency of readily available nutrients in hardwood sawdust as the main substrate, achieving a dual improvement in mycelial growth rate and biomass. In addition, Wang et al. [25] found in their research on *Lentinus edodes* cultivation that the compound use of corn flour with corn cob powder and corn straw powder can replace a portion of hardwood sawdust while still ensuring or even improving mycelial growth and fruiting body yield. This further proves the compatibility and superiority of corn flour as an auxiliary substrate in the hardwood sawdust-based cultivation system, and also provides ideas for the raw material diversification and cost control of the cultivation substrate for *F. luteovirens*.

A substrate–water ratio of 1:1.6 showed the best performance, with significantly leading growth in 30 days and stable growth throughout the entire period. Low water content (≤1:0.8) caused water stress, while high water content (≥1:2.4) resulted in hypoxia. In contrast, the ratio of 1:1.6 precisely balanced moisture and air permeability. This optimal substrate-to-water ratio is consistent with the report by Liu et al. [13] that the mycelial growth of *Agaricus balchaschensis* is optimal at a substrate-to-water ratio of 1:1.5–1:1.7, and aligns highly with the conclusions of similar studies in the edible fungus cultivation field, thus confirming that a substrate-to-water ratio of 1:1.6 is a universally optimal moisture ratio suitable for the growth of various edible and medicinal fungi. Zhang et al. [30] investigated the substrate-to-water ratio for *Cordyceps militaris* cultivation using wheat as the main substrate and found that a ratio of 1:1.6 maximized the fresh weight, density, biological efficiency and substrate utilization rate of *Cordyceps militaris* stromata, while shortening the production cycle to 56 days—the shortest among all experimental groups. This ratio not only provides sufficient water supply for mycelial growth and stromal differentiation of *Cordyceps militaris*, but also avoids the reduction in substrate air permeability caused by excessive water, effectively reduces the risk of contaminating microorganism infestation, and ensures the efficient progress of mycelial respiratory metabolism and nutrient transport. Wang et al. [31] also verified in their research on *Pleurotus ostreatus* cultivation that when a corn cob-based substrate adopted a substrate-to-water ratio range of 1:1.6–1.7, the mycelial colonization speed, substrate colonization ability and subsequent fruiting characteristics were significantly superior to those of other ratios. Among them, 1:1.6 was the core optimal value in this range, which could provide a suitable water environment for *Pleurotus ostreatus* mycelia and promote the mycelial decomposition and utilization of cellulose and hemicellulose in corn cobs. The growth advantage of *F. luteovirens* at a substrate-to-water ratio of 1:1.6 observed in this study echoes the research laws of the above-mentioned edible fungi, all verifying that a substrate-to-water ratio of 1:1.6 can precisely balance the water supply and pore structure of the cultivation substrate: low water content (≤1:0.8) exposes the mycelia of *F. luteovirens* to water stress, hinders cellular metabolism and nutrient absorption, and leads to a significant reduction in germination and growth rates; high water content (≥1:2.4) fills the pores between the cultivation substrate particles, causes substrate hypoxia and inhibits the aerobic respiration of mycelia, while increasing water activity and inducing the propagation of contaminating microorganisms. In contrast, the ratio of 1:1.6 precisely constructs a microenvironment with sufficient water and good air permeability for the mycelial growth of *F. luteovirens*. It not only meets the basic water demand during mycelial growth, but also ensures smooth gas exchange, promoting the rapid colonization, substrate colonization and extension of mycelia—this is the core reason why this ratio has become a universally optimal value for the cultivation of various edible and medicinal fungi.

For substrate compactness, the optimal condition was a substrate surface height of 12–12.5 cm (bulk density: 1.10–1.15 g/cm^3^, porosity: 60.6–63.3%), with a total mycelial growth of nearly 23 mm. Excessively compact substrates (≤11 cm) led to hypoxia, while excessively loose substrates (≥13.5 cm) reduced water retention. This range provided a suitable physical environment for mycelial colonization and extension by regulating the dynamic balance between air permeability and water retention of the culture substrate [32].

Based on the single-factor screening results, the Box–Behnken model was used for synergistic optimization of three factors (main substrate, substrate–water ratio, and substrate compactness). The constructed quadratic regression model exhibited extremely high fitting degree (R^2^ = 0.9920, *p* = 0.0008) with an insignificant lack-of-fit term (*p* = 0.7475), which could accurately describe the effects of the three factors on mycelial growth yield. Analysis of variance (ANOVA) showed that all three factors, as well as all interaction terms and quadratic terms, were extremely significant (*p* < 0.01). Among them, the interaction between substrate–water ratio and substrate compactness (BC) was the most significant (*p* = 0.0009), which is consistent with the mechanism analysis of single-factor experiments: the substrate–water ratio determines the moisture content of the substrate, while substrate compactness determines the pore structure. The two factors synergistically regulate the moisture–air permeability balance, exerting a more critical impact on mycelial growth [33].

Response surface analysis further revealed the interaction rules among various factors. The optimized formula was determined as follows: main substrate 76.002%, substrate–water ratio 1:1.721, and substrate compactness 12.845 cm. The actual measured mycelial growth yield of 28.75 mm was highly consistent with the predicted value (28.012 mm), verifying the practicality and reproducibility of the formula.

The results of this study have clear practical guiding value. By precisely regulating nutritional supply and the physical environment, the optimized culture substrate formula significantly improved the mycelial growth rate and yield of *F. luteovirens*, providing quantifiable technical parameters for its large-scale artificial cultivation. Meanwhile, the established technical process of “preliminary screening—single-factor optimization—response surface synergistic optimization” can serve as a reference for the development of culture substrates for other rare plateau fungi, contributing to the protection and sustainable utilization of plateau biological resources.

### Study Limitations

This study also has certain limitations. Firstly, the experiments were based on a test-tube culture system, which differs from practical production modes such as bag cultivation and bottle cultivation. Factors like substrate packing density and aeration conditions may affect the applicability of the formula, requiring further pilot-scale verification. Secondly, the study did not involve factors such as a culture substrate fermentation process, pH adjustment, and trace element addition, nor did it focus on the impact of the formula on fruiting body formation and quality.

Future research can focus on the following directions: Firstly, targeting practical cultivation modes (e.g., bag cultivation and bottle cultivation) and optimizing the substrate packing process and fermentation conditions to enhance the scenario adaptability of the formula. Secondly, introduce variables such as pH value, fermentation degree, and active additives to construct a more comprehensive multi-factor optimization model. Thirdly, investigate the effects of the formula on fruiting body yield, amino acid content, and bioactive component accumulation, achieving synergistic optimization of “mycelial growth—fruiting body quality”. Fourthly, conduct large-scale pilot experiments based on the optimized formula to evaluate cost feasibility and production stability, promoting the industrialization implementation of artificial cultivation of *F. luteovirens*.

## 5. Conclusions

This study identified the optimal combination of cultivation substrate parameters for the mycelial growth of *F. luteovirens*: hardwood sawdust as the main substrate and corn flour as the auxiliary substrate, and determined a substrate-to-water ratio of 1:1.6–1.7 and a compactness of 12–12.5 cm as the key physical conditions. The formula optimized via response surface methodology significantly increased the mycelial growth (by approximately 92% compared with the basic formula), and the predicted values of the model were highly consistent with the measured values (R^2^ = 0.9920), which verified the reliability of the optimization method.

This study fills the gap in the systematic research on the domestication and cultivation substrates of *F. luteovirens*, and provides key technical parameters for the mycelial growth stage of its artificial cultivation. The optimized cultivation substrate formula can serve as a basic framework for large-scale cultivation, yet its applicability in actual production (e.g., bag cultivation, bottle cultivation) and its impact on fruiting body formation need to be further verified. Future research should focus on the following directions: (1) verifying the adaptability of the formula in bag/bottle cultivation systems; (2) investigating the effects of additional factors such as cultivation substrate fermentation processes, pH values and trace elements; (3) carrying out the whole-chain optimization from mycelial growth to the yield and quality of fruiting bodies.

## Figures and Tables

**Table 1:** Preliminary screening formulas of cultivation substrate.

Serial Number	Components and Proportions
1	Mixed sawdust 75%, wheat bran 20%, potassium dihydrogen phosphate 3%, gypsum 1%, lime 1%
2	Mixed sawdust 63%, cottonseed hulls 20%, wheat bran 12%, corn flour 2%, lime 2.5%, calcium superphosphate 0.5%
3	Mixed sawdust 50%, cottonseed hulls 40%, wheat bran 7%, lime 2.5%, calcium superphosphate 0.5%
4	Mixed sawdust 39%, corn cob 39%, wheat bran 20%, calcium carbonate 2%
5	Mixed sawdust 30%, cottonseed hulls 25%, corn cob 18%, wheat bran 15%, corn flour 5%, soybean meal 5%, calcium superphosphate 1%, gypsum 1%
6	Powdered mixed sawdust 75%, corn flour 23%, gypsum 1%, lime 1%
7	Cottonseed hulls 38%, wheat bran 32%, mixed sawdust 25%, corn flour 3%, light calcium carbonate 1.5%, calcium superphosphate 0.5%
8	Cottonseed hulls 60%, mixed sawdust 22%, wheat bran 10%, corn flour 5%, gypsum 1%, lime 1%, calcium superphosphate 1%
9	Cottonseed hulls 52%, corn cob 25%, mixed sawdust 6%, wheat bran 10%, corn flour 5%, lime 1.5%, calcium superphosphate 0.5%
10	Cottonseed hulls 75%, wheat bran 20%, gypsum 1%, lime 1%, humus 3%
11	Cottonseed hulls 80%, rice straw 10%, wheat bran 8%, lime 2%
12	Cottonseed hulls 75%, wheat bran 20%, gypsum 1%, phosphate fertilizer 1%, humus 3%
13	Cottonseed hulls 75%, corn cob 15%, wheat bran 8%, lime 2%
14	Rice straw 85%, wheat bran 10%, gypsum 3%, calcium magnesium phosphate fertilizer 2%
15	Rice straw 30%, mixed sawdust 15%, cottonseed hulls 20%, powdered dry cow dung 15%, wheat bran 18%, lime 2%
16	Rice straw 30%, cottonseed hulls 10%, powdered dry cow dung 17%, corn cob 25%, wheat bran 10%, corn flour 5%, lime 2.5%, calcium superphosphate 0.5%
17	Rice straw 55.4%, cow dung 36%, calcium superphosphate 0.8%, gypsum 1.4%, soybean cake powder 2.2%, light calcium carbonate 1.1%, urea 0.8%, ammonium bicarbonate 0.8%, lime 1.5%
18	Corn cob 76.8%, corn flour 21.2%, gypsum 1%, lime 1%
19	Corn cob 71.2%, powdered dry cow dung 26.8%, gypsum 1%, lime 1%
20	Corn flour 49%, vermiculite 49%, gypsum 1%, lime 1%
21	*Agaricus bisporus* secondary fermented substrate
22	*Flammulina filiformis* spent mushroom substrate
23	*Flammulina filiformis* fermented scratching substrate
24	Fermented *Volvariella volvacea* spent mushroom substrate
25	*Pleurotus eryngii* spent mushroom substrate
26	*Hypsizygus marmoreus* spent mushroom substrate

**Table 4:** Experimental factors and levels of Box–Behnken design.

Factor	Code		Level		
	Name	Unit	−1	0	1
A	Main Substrates	%	63	73	83
B	Substrate–Water Ratio	1	1:1.4	1:1.6	1:1.8
C	Compactness	cm	11	12	13

**Table 5:** Culture results of 26 preliminary screening cultivation substrate formulas.

Formula	Germination Status	Colonization/Substrate Utilization Status	Mycelial Growth Vigor
1	Germinated after 2 days, good consistency	Colonized after 10 days, substrate utilized but growth too slow	White, relatively dense, neat edges (+++)
2	Germinated after 2 days, good consistency	No colonization or substrate utilization, gradually died after 10 days	White, sparse (+)
3	Germinated after 3 days, good consistency	No colonization or substrate utilization, gradually died after 10 days	White, sparse (+)
4	Germinated after 2 days, good consistency	Colonized after 25 days, substrate utilized	White, dense, irregular edges (++++)
5	Germinated after 2 days, relatively inconsistent	Colonized after 10 days, substrate utilized with continuous growth	White, dense, neat edges (++++)
6	Germinated after 2 days, relatively inconsistent	Colonized after 15 days, substrate utilized	White, relatively dense, neat edges (+++)
7	Germinated after 3 days, good consistency	Colonized after 10 days, substrate utilized	White, relatively sparse, irregular edges (++)
8	Germinated after 3 days, good consistency	No colonization or substrate utilization, gradually died	White, sparse (+)
9	Germinated after 3 days, inconsistent	No colonization or substrate utilization, gradually died after 20 days	White, sparse (+)
10	Germinated after 4 days, good consistency	No colonization or substrate utilization, gradually died after 20 days	White, relatively sparse (++)
11	Germinated after 4 days, inconsistent	No colonization or substrate utilization, gradually died after 10 days	White, sparse (+)
12	Germinated after 3 days, good consistency	Colonized after 20 days, substrate utilized	White, relatively sparse, neat edges (++)
13	Germinated after 4 days, inconsistent	No colonization or substrate utilization, gradually died after 10 days	White, sparse (+)
14	Germinated after 3 days, good consistency	No colonization or substrate utilization, gradually died after 12 days	Contaminated
15	Germinated after 4 days, inconsistent	No colonization or substrate utilization, gradually died after 10 days	White, sparse (+)
16	No germination	-	-
17	No germination	-	-
18	Germinated after 3 days, inconsistent	No colonization or substrate utilization, gradually died after 10 days	White, relatively sparse (++)
19	Germinated after 3 days, inconsistent	No colonization or substrate utilization, gradually died after 10 days	White, sparse (+)
20	Germinated after 3 days, inconsistent	No colonization or substrate utilization, gradually died after 20 days	White, relatively dense (+++)
21	Germinated after 3 days, inconsistent	No colonization or substrate utilization, gradually died after 15 days	White, sparse (+)
22	Germinated after 4 days, good consistency	No colonization or substrate utilization, gradually died after 15 days	White, relatively dense (+++)
23	Germinated after 3 days, inconsistent	No colonization or substrate utilization, gradually died after 5 days	Mycelial blocks browned (+)
24	Germinated after 4 days, inconsistent	No colonization or substrate utilization, gradually died after 5 days	Mycelial blocks browned, sparse (+)
25	Germinated after 3 days, inconsistent	Colonized after 10 days, substrate utilized	White, initially sparse, then gradually dense, relatively neat edges (+++)
26	Germinated after 4 days, inconsistent	Colonized after 10 days, substrate utilized	White, initially sparse, then gradually dense, irregular edges (+++)

Note: + indicates mycelia are white and sparse; ++ indicates mycelia are white and relatively sparse; +++ indicates mycelia are bright white and relatively dense; ++++ indicates mycelia are bright white and dense.

**Table 6:** Average mycelial growth rate in different main substrate formulas.

Formula	Average Mycelial Growth Rate (mm/d)				Significance Level (*p* < 0.5)				Mycelial Growth Vigor
	15 d	20 d	25 d	30 d	15 d	20 d	25 d	30 d	
CK	0.250 ± 0.063	0.450 ± 0.100	0.600 ± 0.163	0.400 ± 0.163	bc	CD	bcde	ABC	++
1	0.268 ± 0.053	0.650 ± 0.191	1.000 ± 0.163	0.500 ± 0.258	ab	ABC	a	AB	++++
2	0.100 ± 0.035	0.350 ± 0.100	0.400 ± 0.163	0.200 ± 0.000	d	D	e	C	+
3	0.218 ± 0.035	0.850 ± 0.191	0.550 ± 0.191	0.500 ± 0.115	bc	A	cde	AB	+++
4	0.218 ± 0.035	0.350 ± 0.100	0.450 ± 0.100	0.250 ± 0.100	bc	D	de	BC	+
5	0.315 ± 0.030	0.350 ± 0.100	0.450 ± 0.100	0.250 ± 0.100	a	D	de	BC	++
6	0.268 ± 0.053	0.700 ± 0.115	0.850 ± 0.252	0.550 ± 0.252	ab	AB	ab	A	++++
7	0.200 ± 0.000	0.550 ± 0.191	0.750 ± 0.191	0.450 ± 0.191	c	BCD	abc	ABC	++
8	0.253 ± 0.035	0.700 ± 0.115	0.650 ± 0.191	0.450 ± 0.100	abc	AB	bcde	ABC	++++
9	0.270 ± 0.000	0.700 ± 0.115	0.700 ± 0.200	0.450 ± 0.100	ab	AB	bcd	ABC	+++

Note: Different letters in the table indicate significant differences among treatments (*p* < 0.05); + indicates mycelia are white and sparse; ++ indicates mycelia are white and relatively sparse; +++ indicates mycelia are bright white and relatively dense; ++++ indicates mycelia are bright white and dense.

**Table 7:** Average mycelial growth rate in different auxiliary material formulas.

Formula	Average Mycelial Growth Rate (mm/d)				Significance Level (*p* < 0.5)				Mycelial Growth Vigor
	15 d	20 d	25 d	30 d	15 d	20 d	25 d	30 d	
CK	0.715 ± 0.083	0.850 ± 0.300	0.750 ± 0.300	0.550 ± 0.300	ab	AB	ab	B	+++
1	0.618 ± 0.067	0.650 ± 0.191	0.550 ± 0.191	0.500 ± 0.115	bc	B	abc	BC	++
2	0.835 ± 0.040	1.050 ± 0.191	0.850 ± 0.342	0.850 ± 0.100	a	A	a	A	++++
3	0.500 ± 0.085	0.200 ± 0.000	0.250 ± 0.100	0.200 ± 0.000	c	C	c	C	+
4	0.750 ± 0.150	1.000 ± 0.163	0.650 ± 0.300	0.500 ± 0.115	ab	A	abc	BC	+++
5	0.675 ± 0.101	0.600 ± 0.163	0.400 ± 0.163	0.450 ± 0.252	bc	B	bc	BC	+++
6	0.715 ± 0.030	0.800 ± 0.163	0.800 ± 0.283	0.700 ± 0.258	ab	AB	ab	AB	++

Note: Different letters in the table indicate significant differences among treatments (*p* < 0.05); + indicates mycelia are white and sparse; ++ indicates mycelia are white and relatively sparse; +++ indicates mycelia are bright white and relatively dense; ++++ indicates mycelia are bright white and dense.

**Table 8:** Average mycelial growth rate in different substrate–water ratio formulas.

Substrate–Water Ratio	Average Mycelial Growth Rate (mm/d)				Significance Level (*p* < 0.5)				Mycelial Growth Vigor
	15 d	20 d	25 d	30 d	15 d	20 d	25 d	30 d	
1:0.5	0.400 ± 00.000	0.400 ± 0.000	0.450 ± 0.100	0.400 ± 0.000	h	G	gh	F	++
1:0.6	0.400 ± 0.000	0.600 ± 0.163	0.400 ± 0.000	0.500 ± 0.115	h	EF	h	DEF	++
1:0.7	0.548 ± 0.035	0.800 ± 0.000	0.600 ± 0.000	0.550 ± 0.100	efg	CD	efgh	CDEF	+
1:0.8	0.533 ± 0.053	0.750 ± 0.100	0.550 ± 0.100	0.600 ± 0.000	fg	DE	fgh	BCDEF	++++
1:0.9	0.515 ± 0.083	0.750 ± 0.100	0.700 ± 0.115	0.700 ± 0.115	g	DE	cdefg	ABCD	++++
1:1.0	0.600 ± 0.000	0.750 ± 0.100	0.650 ± 0.100	0.750 ± 0.100	defg	DE	defg	ABC	++++
1:1.1	0.635 ± 0.040	0.750 ± 0.100	0.850 ± 0.100	0.650 ± 0.100	cde	DE	abcde	ABCDE	+++
1:1.2	0.653 ± 0.035	0.900 ± 0.115	0.700 ± 0.115	0.750 ± 0.100	cd	ABCD	cdefg	ABC	++++
1:1.3	0.670 ± 0.000	0.850 ± 0.100	0.700 ± 0.200	0.650 ± 0.100	cd	BCD	cdefg	ABCDE	++++
1:1.4	0.618 ± 0.067	0.850 ± 0.100	0.850 ± 0.100	0.550 ± 0.300	def	BCD	abcde	CDEF	+++
1:1.5	0.635 ± 0.040	0.600 ± 0.000	0.500 ± 0.115	0.650 ± 0.100	cde	EF	gh	ABCDE	+++
1:1.6	0.965 ± 0.040	0.950 ± 0.100	0.700 ± 0.115	0.850 ± 0.100	a	ABC	cdefg	A	+++
1:1.7	0.933 ± 0.094	0.900 ± 0.115	0.700 ± 0.115	0.800 ± 0.000	a	ABCD	cdefg	AB	+++
1:1.8	0.718 ± 0.099	1.050 ± 0.100	0.800 ± 0.231	0.650 ± 0.252	c	A	bcdef	ABCDE	++
1:1.9	0.618 ± 0.035	0.550 ± 0.100	0.900 ± 0.200	0.800 ± 0.000	def	F	abcd	AB	++
1:2.0	0.818 ± 0.148	0.850 ± 0.100	1.050 ± 0.191	0.650 ± 0.191	b	BCD	a	ABCDE	++
1:2.1	0.815 ± 0.083	0.950 ± 0.191	0.950 ± 0.443	0.550 ± 0.379	b	ABC	abc	CDEF	+++
1:2.2	0.670 ± 0.000	1.000 ± 0.000	0.900 ± 0.115	0.700 ± 0.115	cd	AB	abcd	ABCD	+
1:2.3	0.670 ± 0.000	1.000 ± 0.000	1.000 ± 0.000	0.750 ± 0.100	cd	AB	ab	ABC	+
1:2.4	0.515 ± 0.030	0.500 ± 0.115	0.450 ± 0.100	0.450 ± 0.100	g	FG	gh	EF	+
1:2.5	No germination				-				-
1:2.6	No germination				-				-

Note: Different letters in the table indicate significant differences among treatments (*p* < 0.05); + indicates mycelia are white and sparse; ++ indicates mycelia are white and relatively sparse; +++ indicates mycelia are bright white and relatively dense; ++++ indicates mycelia are bright white and dense.

**Table 9:** Average mycelial growth rate in different substrate compactness formulas.

Substrate Surface Height (cm)	Average Mycelial Growth Rate (mm/d)				Significance Level (*p* < 0.5)				Mycelial Growth Vigor
	15 d	20 d	25 d	30 d	15 d	20 d	25 d	30 d	
10	0.683 ± 0.062	0.800 ± 0.000	0.700 ± 0.200	0.600 ± 0.163	abc	AB	ab	A	++++
10.5	0.618 ± 0.035	0.700 ± 0.200	0.650 ± 0.100	0.600 ± 0.283	cd	AB	ab	A	+
11	0.565 ± 0.040	0.900 ± 0.115	0.500 ± 0.200	0.650 ± 0.191	d	A	b	A	++
11.5	0.618 ± 0.067	0.850 ± 0.191	0.700 ± 0.258	1.100 ± 1.013	cd	AB	ab	A	+++
12	0.700 ± 0.035	0.900 ± 0.115	0.800 ± 0.163	0.850 ± 0.100	ab	A	a	A	++++
12.5	0.715 ± 0.030	0.900 ± 0.115	0.850 ± 0.100	0.750 ± 0.100	a	A	a	A	+++
13	0.700 ± 0.035	0.800 ± 0.163	0.750 ± 0.252	0.900 ± 0.115	ab	AB	ab	A	++
13.5	0.700 ± 0.035	0.800 ± 0.000	0.850 ± 0.100	0.800 ± 0.163	ab	AB	a	A	+++
14	0.635 ± 0.040	0.650 ± 0.100	0.500 ± 0.115	0.550 ± 0.100	bc	B	b	A	+

Note: Different letters in the table indicate significant differences among treatments (*p* < 0.05); + indicates mycelia are white and sparse; ++ indicates mycelia are white and relatively sparse; +++ indicates mycelia are bright white and relatively dense; ++++ indicates mycelia are bright white and dense.

**Table 10:** Corresponding bulk density and porosity results for optimal substrate compactness.

Compactness/cm	Average Total Weight After Saturated Water Absorption/g	Average Total Weight After Oven-Drying/g	Bulk Density/(g·cm^−3^)	Porosity/%
12	54.29	34.97	1.15	63.3%
12.5	54.37	35.1	1.10	60.6%

**Table 11:** Response surface design and experimental results.

Number	A	B	C	Total Mycelial Growth/mm
1	63	1:1.4	12	20
2	83	1:1.4	12	18.7
3	63	1:1.8	12	18.8
4	83	1:1.8	12	25.3
5	63	1:1.6	11	19.5
6	83	1:1.6	11	(eliminate)
7	63	1:1.6	13	18
8	83	1:1.6	13	26.5
9	73	1:1.4	11	20.5
10	73	1:1.8	11	17.5
11	73	1:1.4	13	18.2
12	73	1:1.8	13	26.2
13	73	1:1.6	12	26.25
14	73	1:1.6	12	25.5
15	73	1:1.6	12	27

**Table 12:** Results of analysis of variance (ANOVA).

Source	Sum of Squares	df	Mean Square	F-Value	*p*-Value
Model	186.91	9	20.77	55.20	0.0008
A	11.21	1	11.21	29.80	0.0055
B	13.52	1	13.52	35.93	0.0039
C	16.33	1	16.33	43.41	0.0027
AB	15.21	1	15.21	40.43	0.0031
AC	14.05	1	14.05	37.33	0.0036
BC	30.25	1	30.25	80.40	0.0009
A^2^	25.23	1	25.23	67.06	0.0012
B^2^	21.07	1	21.07	55.99	0.0017
C^2^	27.00	1	27.00	71.76	0.0011
Residual	1.51	4	0.3763		
Lack of Fit	0.3800	2	0.1900	0.3378	0.7475
Pure Error	1.13	2	0.5625		
Cor Total	188.41	13			

## Data Availability

No data was used for the research described in the article.
